# Non-coding RNA-mediated endothelial-to-mesenchymal transition in human diabetic cardiomyopathy, potential regulation by DNA methylation

**DOI:** 10.1186/s12933-023-02039-4

**Published:** 2023-11-03

**Authors:** Eric Wang, Shali Chen, Honglin Wang, Tori Chen, Subrata Chakrabarti

**Affiliations:** Department of Pathology and Laboratory Medicine, Western University, Dental Science Building Room 4033, 1151 Richmond St, London, ON N6A 3K7 Canada

**Keywords:** Diabetic cardiomyopathy, Endothelial-to-mesenchymal transition, Epigenetics, Long non-coding RNA, microRNA, Promoter methylation

## Abstract

**Aims:**

Diabetic cardiomyopathy (DCM) is a major complication of diabetes and a risk factor for cardiovascular disease. Endothelial dysfunction is central to DCM, and endothelial-to-mesenchymal transition (EndMT) is a key form of endothelial dysfunction in diabetes. EndMT in DCM has been well-studied in model systems and has been found to be epigenetically regulated by non-coding RNAs (ncRNAs). However, EndMT in DCM and its associated epigenetic changes need further characterization in human patients. It is also not known if ncRNAs are affected by changes in DNA methylation in DCM. This study aims to confirm in human hearts, the findings from animal and cell studies, and potentially provide novel insight into interactions between DNA methylation and ncRNAs in EndMT in DCM.

**Methods and results:**

Heart tissues were collected from autopsy patients, fixed in formalin, and embedded in paraffin. Thin sections from paraffin-embedded tissues were used for histology and immunofluorescence analyses, where we confirmed that diabetic patients showed increased cardiac fibrosis that EndMT had occurred. Tissue curls from the paraffin-embedded tissues were used for RT-qPCR and methylation analyses. RT-qPCR quantitatively showed that EndMT occurs in the hearts of diabetics, and that EndMT in human hearts corresponded to changes in key ncRNAs. Methylation analysis showed that some of the EndMT-related ncRNAs were regulated by DNA promoter methylation, while others may be regulated through different epigenetic mechanisms.

**Conclusions:**

We show that EndMT is a relevant pathological process in human hearts during DCM, and that its occurrence coincides with changes in relevant ncRNAs. We further find that interplay between DNA methylation and certain ncRNAs involved in the regulation of EndMT may contribute to the observed changes in ncRNA expression. These findings reinforce the role of EndMT in patients afflicted with DCM and underscore the complexities and importance of the interactions between different facets of epigenetic regulation.

**Supplementary Information:**

The online version contains supplementary material available at 10.1186/s12933-023-02039-4.

## Background

Diabetes is a significant cause of death globally and is associated with an up to fourfold increase in cardiovascular mortality [1]. Diabetic cardiomyopathy (DCM), a major cardiac complication of diabetes, is a significant and independent risk factor for cardiovascular disease [2–4]. DCM involves left ventricular hypertrophy as a result of cardiomyocyte hypertrophy, interstitial and perivascular fibrosis, myocyte death, and thickening of the myocardial capillary basement membranes [2–4]. These changes are the direct result of hyperglycemia-induced damage in diabetes and are often amplified by comorbidities in type 2 diabetics [2–4]. Hyperglycemia includes oxidative stress and activates multiple interconnected and harmful pathways (such as the formation of advanced glycation end-products), resulting in cellular damage and tissue inflammation [2–4]. Compensatory responses are also activated, leading to structural changes and tissue remodelling [2–4].

Central to the pathogenesis of DCM are endothelial cells (ECs), which are the most abundant cell type in the heart, and among the first to experience hyperglycemic damage [5–7]. ECs can undergo a process known as endothelial-to-mesenchymal transition (EndMT), a dynamic and highly regulated process by which ECs transdifferentiate into mesenchymal-like cells [8–11]. ECs that undergo EndMT acquire morphological and phenotypical characteristics of mesenchymal cells; exhibiting increased expressions of extracellular matrix proteins, decreased expressions of endothelial junctional proteins, and gaining a spindle-like shape and increased motility [8–11]. EndMT plays a crucial role in early heart development, contributing to the formation of the cardiac cushions, which give rise to the heart valves and septa [11, 12]. EndMT also contributes to the pathogenesis of cardiovascular diseases, cancer, and fibrosis [10–14]. EndMT is regulated by signalling pathways such as inflammatory signalling, transforming growth factor β (TGF-β), Notch, and Wnt, which can become dysregulated in disease states [14, 15]. Hyperglycemia is also a direct driver of EndMT; high glucose-induced generation of reactive oxygen species and activation of intracellular pathways trigger pro-EndMT changes in gene expression [16]. Furthermore, hyperglycemia also induces inflammation and TGF-β signalling, further promoting EndMT [8, 15, 17, 18]. We and others have shown that EndMT plays a significant role in DCM, and diabetic cardiac fibrosis [8–10, 19].

EndMT-derived mesenchymal cells become committed to the mesenchymal phenotype through alterations in epigenetic regulation, which perpetuate the mesenchymal characteristics in progeny cells even in the absence of the inciting stimulus [10, 20]. Epigenetic regulation describes the mechanisms of heritable phenotypical change, absent modifications to the genomic sequence [20]. The main facets of epigenetic regulation include histone modifications, DNA methylation, and gene regulation by non-coding RNAs (ncRNAs) [20, 21]. DNA methylation involves the covalent attachment of methyl groups to nucleotides by DNA methyltransferases (DNMTs)—most commonly to the 5th carbon of cytosine residues in a CpG pair—and generally results in transcriptional inhibition; methyl groups can also be removed by DNA demethylases, which is typically associated with upregulation of the gene [21, 22]. Gene regulation by ncRNAs involve potentiation or inhibition of protein-coding genes by transcribed elements that do not encode proteins [21, 23]. The two main classes of ncRNAs are long non-coding RNAs (lncRNAs) and microRNAs (miRNAs). lncRNAs are ncRNAs greater than 200 nucleotides in length, they can act through cis- or trans-regulatory mechanisms, regulating nearby or distant genes respectively [24, 25]. Due to their size, lncRNAs can form complex tertiary structures and interact with a variety of RNA-binding proteins, serving as scaffolds, guides, or decoys for protein complexes, leading to activation or repression of target loci [24, 25]. miRNAs are small ncRNAs roughly 22 nucleotides in length, they mediate translational silencing or degradation of target transcripts through complementarity with the 3ʹ untranslated regions of mRNAs [26, 27]. Histone modifications involve covalent changes to histone proteins—such as methylation, acetylation, phosphorylation, or citrullination—leading to activation or repression of the associated DNA regions [21, 28]. In general, DNA and histone modifications influence transcriptional regulation by directly modifying the chromatin, whereas ncRNAs can participate in transcriptional, post-transcriptional, and translational levels. There is reciprocal regulation among the three facets of epigenetic regulation, the balance of which determines gene expression [22–24, 28, 29].

Epigenetic regulations have long been recognized to be involved in cardiovascular diseases, and recent evidence have also indicated their importance in diabetes-induced EndMT. Hyperglycemia-induced dysregulation of DNMT3 and the histone deacetylase (HDAC) SIRT1 have been shown to contribute to cardiac dysfunction in diabetic animals [30], and activation of SIRT1 has been shown to improve cardiac outcomes [31]. HDACs 3 and 9 have been shown to induce EndMT in atherosclerotic plaques [32, 33], and DNMT1 has been found to promote EndMT in diabetic retinopathy by hypermethylating the lncRNA MEG334. Recently, miR-132-3p—downregulated in diabetes—has been reported to regulate EndMT in the aorta via regulation of Kruppel-like factor 7 [35]. We have previously identified lncRNA ZFAS1 and miRNAs 9, 146a, and 200b as regulators of fibrosis and EndMT in DCM and other diabetic complications in cell culture and in animal models [8, 9, 18, 36–38]. mir-200b and miR-146a regulate TGF-β and proinflammatory signaling, respectively, and are downregulated in diabetic cardiac Ecs [8, 36, 37]. ZFAS1 and miR-9 form a regulatory axis in DCM, where high glucose induces ZFAS1 upregulation, which targets the polycomb repressive complex 2 (PRC2) to the miR-9 locus, suppressing miR-9 and promoting EndMT and fibrosis [9]. miR-9 suppresses NFKB1 [39], TGFBR2 [40], and lncRNA, MALAT1, which has been shown to participate in inflammation, fibrosis, and EndMT [41–43]. The ZFAS1–PRC2–miR-9–MALAT1 axis exemplifies the interconnected network between epigenetic regulators in DCM. Yet not a lot is known about the interplay between DNA methylation and ncRNAs in human DCM. The purpose of this study was to confirm previous findings from animal studies and investigate potential interactions between DNA methylation and specific ncRNAs in DCM.

## Methods

### Sample collection/acquisition

The study was approved by the Western Health Sciences Research Ethics Board and Lawson Health Research Institute at the University of Western Ontario (London, ON, CAN), and conducted in accordance with the principles of the Declaration of Helsinki. Cardiac tissues were collected at the time of autopsy, as a routine procedure. Briefly, 3 mm thick tissue slices were taken and fixed overnight in 10% neutral buffered formalin and processed for paraffin embedding. Tissues sections were histologically reviewed by pathologists. Formalin fixed paraffin embedded (FFPE) Sects. (5-10μm) of the left ventricles were collected and used for further analysis. The samples were categorized into two groups: non-diabetic, and diabetic. None of the patients have any known history of myocardial infarction. In some patients, DCM was established via clinical investigation (electrocardiogram, echocardiogram). DCM was confirmed via histological examination of cardiac tissues at autopsy. A summary of patient demographic information can be found in Table 1, complete data is available in Additional file 1: Table S1. Given the nature of the availability of autopsy samples, there are differences in parameters such as age and renal function in the patients. However, these differences are not significant, and correlation analysis showed no significant correlations between age/renal function and parameters measured in the current study **(**Additional file 2: Table S2**).** All experiments were performed in a masked fashion.

**Table 1:** Patient demographic data summary

	Non-diabetic	Diabetic	
Number	10	28	
Age	52.4 ± 24.6 (3–80)	63.6 ± 14 (20–84)	p = 0.09
Sex			
Male	4	19	
Female	6	9	
Glycated hemoglobin	N/A	7.4 ± 2.4 (4–15.4)	
Creatinine (µmol/L)	198.5 ± 148 (32–416)	216.6 ± 211.5 (32–912)	p = 0.81
eGFR (mL/min/1.73m^2^)	53.9 ± 14.8 (8.3–122.2)	46.5 ± 6.1 (5.2–140)	p = 0.58

Data presented as mean ± SEM, with the range in parentheses

### Histology

Sections of 5 μm were cut from each block and mounted onto positively charged slides. Sections were deparaffinized and stained with Masson’s trichrome stain. Stained slides were scanned using a slide scanner (Aperio ScanScope), and myocardial structural assessments were done. The presence of focal fibrosis was assessed using an arbitrary scoring system (1 = no fibrosis, 2 = 1 focus of scarring, 3 =  > 1 focus of scarring) as previously described [44]. Final images were exported using the Aperio ImageScope software.

### Immunofluorescence

Sections (5 µm) were deparaffinized, blocked and stained with rabbit anti-CD31 (1:200, ab28364; Abcam) and mouse anti-SM22 (1:200, 60213-1-Ig; Proteintech) antibodies for 1 h at room temperature. The sections were washed and incubated with secondary antibodies (Alexa Fluor 555 goat anti-mouse and Alexa Fluor 488 goat anti-rabbit; Invitrogen) for one hour at 1:200 dilution. Fluorescence was examined on a fluorescent microscope (Olympus BX51; Olympus, Tokyo, Japan). Images were taken and processed using the Infinity 3 camera (Lumenera Corporation, Ottawa, Canada) and its associated software.

### Isolation and purification of total RNA from FFPE tissues

3–5 sections of 10 μm were cut from each sample block and deparaffinized with 1 mL xylene in a 1.5 mL tube overnight. Tubes were centrifuged at 13100 RPM for 2 min at room temperature, the xylene was removed, and 1 mL 100% ethanol was added. Tubes containing ethanol were vortexed for 45 s and centrifuged at full speed for 2 min at room temperature. Ethanol was removed and total RNA was isolated and purified using the RNeasy FFPE Kit (Qiagen) following the manufacturer’s instructions.

### mRNA and lncRNA quantification

cDNA was synthesized using 2 μg of total RNA with the high-capacity cDNA reverse transcription kit (Applied Biosystems). Real time reverse transcription quantitative polymerase chain reaction (RT-qPCR) was performed using the LightCycler 96 instrument (Roche). Each RT-qPCR reaction comprised 10 μL SYBR Advantage qPCR Premix (Clontech), 1 μL each of forward and reverse primers (10 μmol/L), 6 μL H_2_O, and 2 μL cDNA (primer sequences and amplification conditions can be found in Table 2). mRNA and lncRNA levels were quantified via the standard curve method using a serially diluted standard template. mRNA and lncRNA levels of each sample were normalized to an internal control, *ACTB*, in order to account for potential variations in sample quality, reverse transcription efficiencies, or amount of usable template in reaction mixtures.

**Table 2:** Primer sequences and thermocycler settings for RT-qPCR

Target (product size)	Primer	Sequence (5ʹ–3ʹ)	Temperature profiles	
*ACTB* (215)	Forward Reverse	CCTCTATGCCAACACAGTGC CATCGTACTCCTGCTTGCTG	Denaturation Annealing Extension Signal	95 °C for 5 s 55 °C for 10 s 72 °C for 15 s 84 °C for 1 s
*CDH5* (152)	Forward Reverse	CTACCAGCCCAAAGTGTGTG GTGTTATCGTGATTATCCGTGA	Denaturation Annealing Extension Signal	95 °C for 5 s 55 °C for 10 s 72 °C for 15 s 82 °C for 1 s
*COL1A1* (140)	Forward Reverse	GAGGGCCAAGACGAAGACATC CAGATCACGTCATCGCACAAC	Denaturation Annealing Extension Signal	95 °C for 5 s 55 °C for 10 s 72 °C for 15 s 84 °C for 1 s
*IL6* (149)	Forward Reverse	GGGGCTGCTCCTGGTGTTG CTGAGATGCCGTCGAGGATGTA	Denaturation Annealing Extension Signal	95 °C for 5 s 55 °C for 10 s 72 °C for 15 s 80 °C for 1 s
*MALAT1* (119)	Forward Reverse	TCTTAGAGGGTGGGCTTTTGTT CTGCATCTAGGCCATCATACTG	Denaturation Annealing Extension Signal	95 °C for 5 s 55 °C for 10 s 72 °C for 15 s 80 °C for 1 s
*PECAM1* (158)	Forward Reverse	AGACAACCCCACTGAAGACGTCG CCTCTCCAGACTCCACCACCTTAC	Denaturation Annealing Extension Signal	95 °C for 5 s 55 °C for 10 s 72 °C for 15 s 82 °C for 1 s
*TAGLN* (191)	Forward Reverse	GGCAGGCCCCAGTAAAGAAG TGCCAGCCCACCCAGATT	Denaturation Annealing Extension Signal	95 °C for 5 s 55 °C for 10 s 72 °C for 15 s 84 °C for 1 s
*ZFAS1* (91)	Forward Reverse	CAGCGGGTACAGAATGGA TCAGGAGATCGAAGGTTGTAGA	Denaturation Annealing Extension Signal	95 °C for 5 s 55 °C for 10 s 72 °C for 15 s 82 °C for 1 s

An initial denaturation phase was carried out at 95 °C for 2 min. 50 cycles were used for amplification

### Isolation and purification of genomic DNA and DNA bisulfite from FFPE tissues

Sections were cut from FFPE blocks and deparaffinized as described above. QIAamp DNA FFPE tissue Kit (Qiagen) was used for DNA isolation and purification as per the manufacturer’s instructions. EpiTest Plus DNA Bisulfite Kit (Qiagen) was used for bisulfite conversion of DNA according to the manufacturer’s instructions.

### Promoter methylation-specific qPCR

qPCR was performed using the LightCycler 96 system (Roche) as described above, using bisulfite-converted DNA rather than reverse transcribed cDNA. Methylation-specific qPCR primers were designed to target one or more CpG’s of the core promoters in the sense strands of the target ncRNAs (sequences can be found in Table 3). ZFAS1 promoter primers were designed using the MethPrimer program by Li and Dahiya [45]. The miR-9 and miR-200b promoter primers were taken from published papers [46, 47]. Amplification conditions were as follows: activation cycle (95 °C for 10 min), denaturation (95 °C for 1 s), annealing (55 °C for 20 s), extension (72 °C for 15 s). Methylation levels were quantified using the standard curve method, constructed using a serially diluted standard template. [methylation data presented as ratio between methylated and unmethylated, in order to account for potential variations in sample quality, reverse transcription efficiencies, or amount of usable template in reaction mixtures.]

**Table 3:**
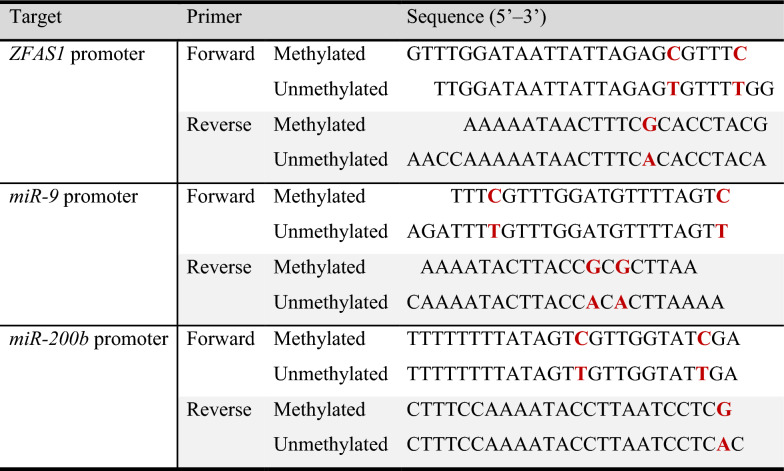
Methylation-specific PCR primers

### Isolation and purification of total miRNA from FFPE tissues

Sections were sectioned and deparaffinized as described above. Taqman miRNA ABC Purification Kit (Thermo Fisher Scientific) was used to isolate and purify total miRNA, following the manufacturer’s instructions.

### miRNA quantification

cDNA was synthesized via reverse transcription using 0.5 μg of total miRNA with the Taqman MicroRNA Reverse Transcription Kit (Thermo Fisher Scientific). TaqMan miR-9, miR-146a, and miR-200b assays (Ambion) were used according to the manufacturer’s instructions to quantify the expressions of miR-9, -146a, and -200b via RT-qPCR. miRNA expressions were normalized to U6 short nuclear RNA, in order to account for potential variations in sample quality, reverse transcription efficiencies, or amount of usable template in reaction mixtures.

### Statistical analysis

Statistical analyses were performed using the open-source software JASP. Independent samples T-tests were performed for gene expressions and promoter methylation levels in diabetic vs non-diabetic samples, and Spearman’s rho test was performed for correlation analysis between age/renal function and genes of interest.

## Results

### Patients with diabetes show increased cardiac fibrosis

**Fig. 1:**
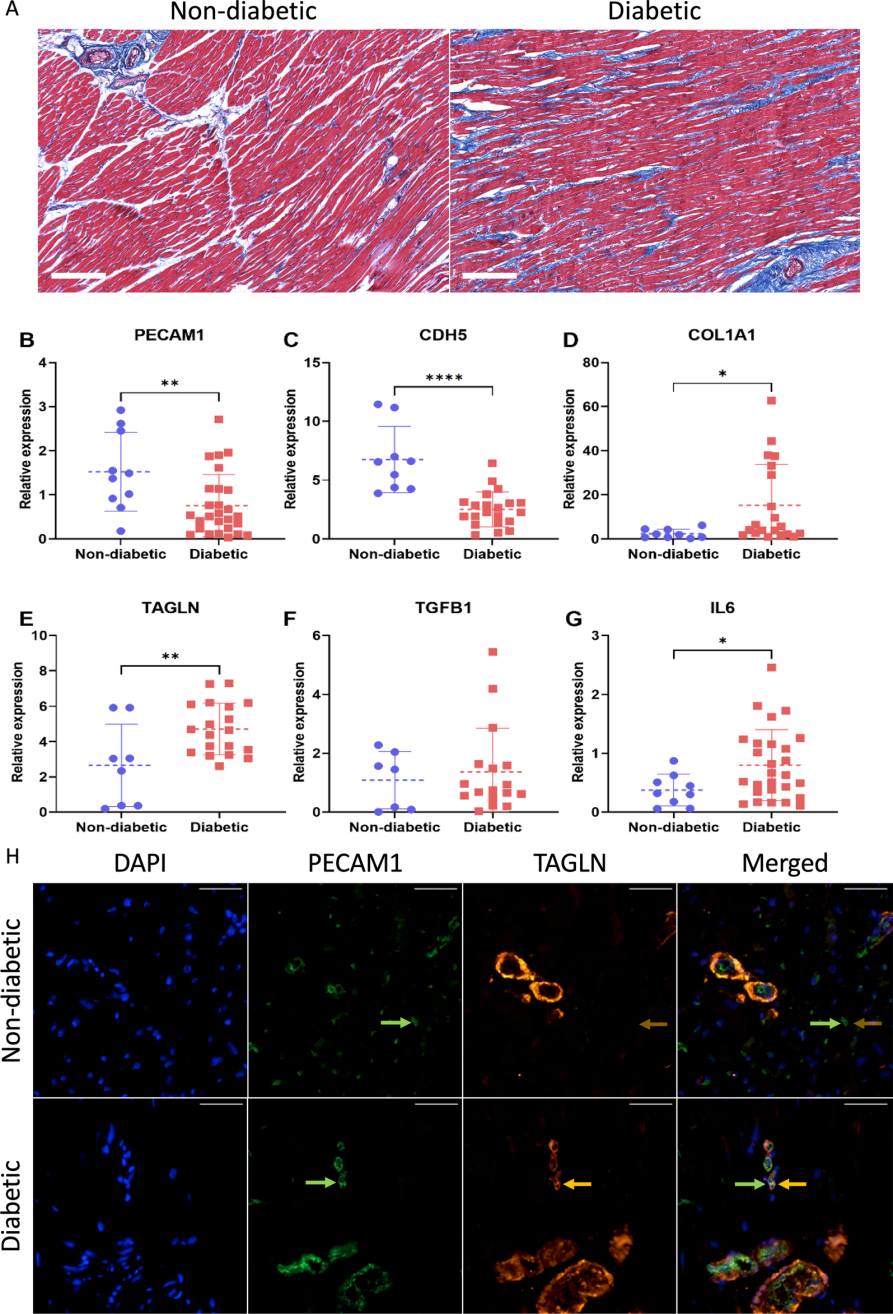
Diabetes is associated with cardiac fibrosis and EndMT in the heart. **A** Trichrome staining shows increased fibrosis around large vessels and in the cardiac interstitium of diabetic patients (scoring 3, in contrast to mostly 1 in non-diabetic individuals). mRNA expressions of endothelial markers **B** *PECAM1* and **C** *CDH5* were significantly decreased, while mRNA expressions of mesenchymal markers D) *COL1A1* and **E** *TAGLN* were significantly higher in diabetic compared to non-diabetic patients. Expressions of pro-EndMT regulators, **F** the profibrotic mediator *TGFB1* and **G** the proinflammatory cytokine *IL6* were also higher in diabetic patients. **H** Immunofluorescence showed that the endothelial marker CD31 (green) was highly expressed in the small vessels in the hearts of non-diabetic patients while SM22 (red) showed minimal immunofluorescence in these vessels, indicating minimal expression. Diabetic patients showed weaker CD31 staining and stronger SM22 staining in the small vessels of the heart, they also showed overlapping red-green fluorescence, reflecting co-localization of the two markers, indicating EndMT. [white bar = 200 μm in trichrome; representative images were chosen; mRNA expressions normalized to *ACTB* mRNA; n = 10 for non-diabetic and n = 28 for diabetic in trichrome and mRNA analyses; data presented as scatter plots with mean ± standard deviation; * = p < 0.05, ** = p < 0.01, *** = p < 0.001, ****p < 0.0001 as determined by Student’s T-test; white bar = 50 μm in fluorescence images; images were uniformly adjusted to reduce background noise; representative images were chosen; n = 7 for non-diabetic and n = 10 for diabetic for fluorescence imaging]

Trichrome staining was done to establish the presence and extent of DCM-associated cardiac fibrosis. As expected, cardiac tissues from non-diabetic patients showed positive staining for collagen in the region immediately surrounding blood vessels and minimal staining elsewhere. Hearts from diabetic patients however, showed robust collagen staining in a larger area around blood vessels, as well as throughout the cardiac interstitium. Using our scoring system, the hearts from non-diabetic individuals showed a score of 1 or occasionally 2, whereas the cardiac tissues from all diabetic individuals showed a fibrosis score of 3 (Fig. 1A).

### Hearts from patients with diabetes demonstrated pathological EndMT

RNA analyses and duo immunofluorescence staining were used to confirm the occurrence of EndMT in human hearts. Heart tissues of diabetic patients showed strong molecular evidence of EndMT. They showed significantly reduced mRNA expressions of endothelial markers *PECAM1* and *CDH5*, and increased mRNA expressions of the mesenchymal marker *TAGLN*, an actin-crosslinking protein found in smooth muscles and fibroblasts, and the matrix protein *COL1A1* (Fig. 1B–E). These tissues also showed an upward trend in mRNA expressions of the profibrotic mediator *TGFB1*, and significant upregulation of the proinflammatory cytokine *IL-6* (Fig. 1E, F) reflecting a pro-fibrotic and proinflammatory environment in the heart, conducive to EndMT. Tissue immunofluorescence showed that altered profiles of endothelial and mesenchymal markers co-localized to microvascular endothelial cells (Fig. 1H). Small vessels in the hearts of non-diabetic patients showed strong CD31 staining (green), with little to no SM22 (red) posivitiy. Small vessels in the hearts of diabetic patients on the other hand, showed reduced CD31 staining and positive staining for SM22. CD31 and SM22 staining co-localized to small vessels, indicating diabetes-induced EndMT.

### Diabetes-induced EndMT is associated with upregulation of specific lncRNAs and downregulation of related miRNAs

**Fig. 2:**
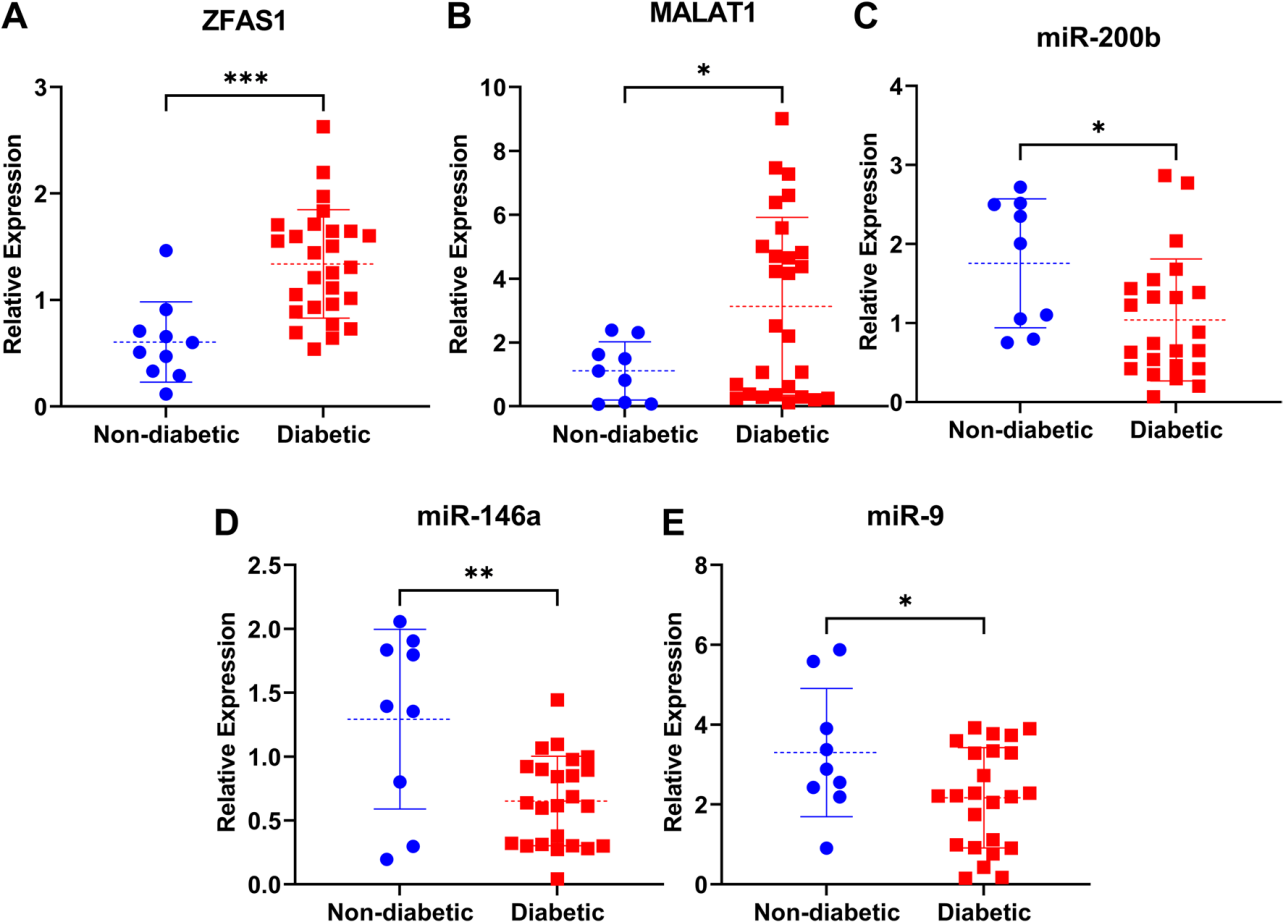
Diabetes is associated with upregulation of pro-EndMT lncRNAs and downregulation of anti-EndMT miRNAs in cardiac tissues. Expressions of anti-EndMT miRNAs **A** miR-9, **B** miR-146a, and **C** miR-200b were significantly lower in the hearts of diabetic compared to non-diabetic patients. Expressions of pro-EndMT lncRNAs **D** *ZFAS1* and **E** *MALAT1* were significantly higher in the hearts of diabetic compared to non-diabetic patients. [miRNA expressions normalized to U6 small nuclear RNA; lncRNA expressions normalized to *ACTB* mRNA; n = 10 for non-diabetic, n = 28 for diabetic; data presented as scatter plots with mean ± standard deviation; * = p < 0.05, ** = p < 0.01, *** = p < 0.001 as determined by Student’s T-test]

Having observed the occurrence of EndMT in the hearts of diabetic patients, next we assessed the changes in ncRNAs of interest. We found that lncRNAs ZFAS1 and MALAT1 were significantly unregulated in the hearts of diabetic patients (Fig. 2D, E). On the other hand, levels of miRs − 9, − 146a, and − 200b were all significantly reduced in the hearts of diabetic patients compared to those of non-diabetic patients (Fig. 2A–C). While this data does not prove a causative relation between the changes in ncRNAs and the occurrence of EndMT, it supports our hypothesis and is supported by findings from animal models.

### Heterogeneity of promoter methylation of ncRNAs in the heart in diabetes

**Fig. 3:**
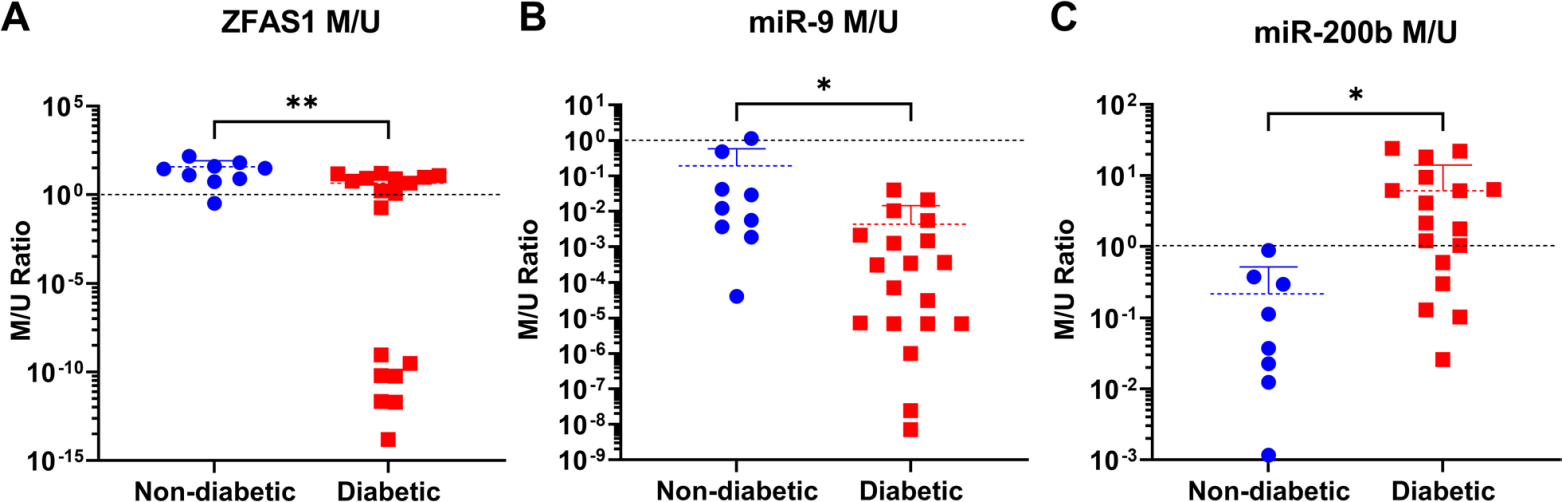
Changes in promotor methylation of ZFAS1, miR-9, and miR-200b in the heart in diabetes. Methylation of **A** ZFAS1 and **B** miR-9 promoters decreased significantly in the heart in diabetes compared to non-diabetic ones, but ZFAS1 promoter maintained an average M/U ratio of greater than 1 (denoted by the dashed line), whereas miR-9 promoter M/U ratio was less than 1 in both non-diabetic and diabetic cohorts. Methylation of **C** miR-200b promoter was significantly increased, with the M/U ratio changing from < 1 to > 1. [lncRNA expressions normalized to *ACTB* mRNA; miRNA expressions normalized to U6 small nuclear RNA; n = 10 for non-diabetic, n = 28 for diabetic; data presented as scatter plots with mean ± standard deviation; * = p < 0.05, ** = p < 0.01 as determined by Student’s T-test]

Methylation analysis showed that ZFAS1 promoter was hypermethylated in the hearts of non-diabetic patients, with a methylated to unmethylated (M/U) ratio greater than 1 (Fig. 3A). Methylation of the ZFAS1 promoter significantly decreased in diabetics—approximately ten-fold—but the M/U ratio remained greater than 1. Methylation of the miR-9 promoter also significantly decreased in the hearts of diabetic compared to non-diabetic patients (Fig. 3B), however, the M/U ratio of the miR-9 promoter was < 1 in both groups. miR-200b promoter methylation was significantly increased in the hearts of diabetics, and its M/U ratio went from < 1 in the non-diabetic group to > 1 in the diabetic group (Fig. 3C).

## Discussion

DCM is a significant risk factor for cardiovascular mortality in patients with diabetes [2–4]. Hyperglycemia causes endothelial dysfunction, smooth muscle alterations, myofibroblast activation, and cardiomyocyte death, leading to structural and functional deficits resulting in heart failure [2–4]. Endothelial dysfunction is perhaps the earliest manifestation of hyperglycemic damage and paves the way for further damage by increasing vascular permeability [5–7]. In this study, we used cardiac tissues from autopsies to investigate one specific manifestation of endothelial dysfunction, EndMT, in DCM. We further examined the expressions and promoter methylation statuses of several ncRNAs of interest as identified by previous in vitro and in vivo studies [8, 9, 37, 38, 48]. We show, for the first time, that cardiac microvascular endothelia in patients with DCM experience pathological EndMT. We confirm in human patients, specific alterations of EndMT-related mechanistic ncRNAs as predicted by studies done in vitro and in vivo. Finally, we report that DNA methylation correlates with, and may contribute to some but not all changes in ncRNAs of interest.

Vascular ECs line the surfaces of the cardiovascular system. Endothelial dysfunction in the form of EndMT has been observed and studied across various cardiovascular pathologies. EndMT has been frequently shown to occur within atherosclerotic plaques—where it contributes to the initiation and progression of atherosclerosis [49–51]. EndMT has also been found to occur in calcific aortic valve disease, where it precedes EC osteogenesis [52, 53], and in diabetes-related aortic stiffening [35]. These instances of EndMT have been shown in both experimental models and in humans [35, 51, 52]. We and others have shown that hyperglycemia-induced EndMT occurs and contributes to DCM both in vitro and in vivo, but these findings have never been recapitulated in human diabetic patients [8, 9, 16, 19, 37]. In animal models of DCM, EC-derived mesenchymal cells facilitate increased deposition of matrix proteins, leading to diabetic cardiac fibrosis [9, 19, 42]. Experimental models of cardiac fibrosis have shown that up to 35% of fibroblasts are of endothelial origin [54, 55]. The occurrence and relevance of hyperglycemia-induced EndMT in DCM is well-supported by basic science, but here we observe the phenomenon in human hearts with DCM. Though we were working with whole heart tissue, rather than isolated endothelial cells, we found significant downregulation of endothelial markers and upregulation of matrix proteins in hearts of diabetic patients. If we were to argue that the upregulation of matrix protein expression was due solely to activation of resident myofibroblasts, it would not account for the downregulation of endothelial markers. Such findings suggest that EndMT is responsible for at least a portion of the observed changes in gene expression. This notion is further supported by double immunofluorescence staining of cardiac tissues, showing co-localized increase in mesenchymal markers and decrease in endothelial markers in the small heart vessels of diabetics.

Given the prevalence of EndMT in cardiovascular pathology, the regulatory mechanisms of pathological EndMT have received a fair amount of attention, though most of it had been focused around atherosclerosis. In cardiovascular pathologies, EndMT is generally understood to be induced by inflammation or by dysregulation of key signalling pathways such as TGF-β, Wnt, and Notch, leading to altered epigenetic regulation and aberrant gene expression [14, 15]. Subsequent mesenchymal characteristics are perpetuated by epigenetic modulators such as DNMTs, HDACs, and ncRNAs [8, 9, 18, 31–39]. Other novel pathways are discovered from time to time, interestingly, deficiency of the circadian locomotor output cycles protein kaput (*CLOCK*) gene in atherosclerotic plaques has been shown to promote EndMT via upregulation of rho-associated coiled-coil-containing protein kinase 1 (*ROCK1*) [56], which had been previously identified as a promoter of glomerular EndMT in diabetic nephropathy [57], and which has been reported to be indirectly regulated by lncRNA ZFAS1 in renal mesangial cells [58].

We have studied the ncRNAs ZFAS1, MALAT1, miR-9, miR-146a and miR-200b in the context of diabetes and hyperglycemia-induced EndMT. As is the case with most other mechanistic studies, we used model systems in which we can easily manipulate the expressions of our candidate regulators to establish causational relations. In this study, we report for the first time, increased expressions of pro-EndMT lncRNAs and decreased expressions of anti-EndMT miRNAs in the hearts of patients with DCM. These observations substantiate our previous in vitro and in vivo investigations and highlight the importance of ncRNAs in hyperglycemia-induced EndMT in DCM. It must be noted however, that because we are using whole cardiac tissue, rather than isolated cardiac ECs, we cannot rule out potential contributions from other cell types of the heart. Despite ECs being the most abundant cell type within the heart, we are not able to conclude that the observed changes in ncRNA expressions correspond solely to hyperglycemic EndMT. For example, while miR-146a and -200b have never been reported to change in cardiomyocytes in response to high glucose, studies have found upregulation of ZFAS1 and MALAT1, and downregulation of miR-9 in high glucose-treated cardiomyocytes [59–61]. Hence it may be possible that ECs account for most of the observed changes in miR-146a and -200b expressions and some but not all changes in ZFAS1, MALAT1, and miR-9 expressions.

The observations of EndMT and altered expressions of related ncRNAs in human hearts with DCM are largely validations of previous findings from cell and animal models [8, 9, 18, 36–38, 42]. In this study, we further take the opportunity to explore other facets of the complex regulation of hyperglycemia-induced EndMT in DCM. While the importance of histone modifications in EndMT have been well-established, little work has been done with DNA methylation, and even less is known about promoter methylation of EndMT-related ncRNAs. This foray into ncRNA promoter methylation in DCM serves as an exploratory investigation to identify important links between DNA methylation, ncRNAs and EndMT.

We find that while the methylation ratio of the ZFAS1 promoter remained greater than 1 in both diabetic and non-diabetic cohorts, promoter methylation significantly decreased in the diabetic group, corresponding to the increase in ZFAS1 expression in the hearts of diabetic patients. On the other hand, miR-200b promoter became significantly hypermethylated in the diabetic group, corresponding to its transcriptional repression in the hearts of diabetics. This finding is contradicted by that of Singh et al., who reported hypomethylation of the miR-200b promoter region in microvascular ECs in response to high glucose [62]. Interestingly, they also reported that miR-200b expression was increased in human microvascular ECs under high glucose treatment [62], which is inconsistent with the findings of other studies involving miR-200b in diabetes [8, 36, 63, 64]. Such differences may result from the use of varying models and conditions, such as different EC types, culture media, glucose concentrations, and duration of high glucose exposure.

Perhaps a slightly more unexpected outcome from the promoter methylation analysis was observed at the miR-9 promoter, where downregulation of miR-9 in diabetes was associated with decreased promoter methylation. Hypomethylation of a promoter is typically associated with increased expression of a gene, yet we have observed the opposite, thus we must consider the wider context of epigenetic regulation of the miR-9 locus under diabetic conditions. We have recently reported that in cardiac ECs, miR-9 is regulated by ZFAS1 through PRC2, a repressive regulator which primarily acts by trimethylating lysine 27 of the histone H3 protein (H3K27me3), causing the associated region to condense into heterochromatin [9, 28]. Studies have shown that H3K27me3 modifications are largely incompatible with DNA methylation [65–67]. Brinkman et al. found in a genome-scale study that CpG-rich promoter regions that were marked with H3K27me3 were exclusively unmethylated65. While others have found that DNA methylation and H3K27me3 act antagonistically toward one another [66, 67], largely resulting in mutually exclusive presence of H3K27me3 or DNA methylation. In this context, it is not extraordinary that miR-9 promoter would be largely unmethylated in the diabetic group, given that we expect to find ZFAS1-guided PRC2-mediated histone methylation of the same locus.

It is important to note that this study is observational in nature and does not establish cause-effect relations between DCM and EndMT, EndMT and ncRNA expressions, or ncRNA expressions and ncRNA promoter methylation. For the first two relations, we have previously established the cause-and-effect relations in culture and in animal models, so these findings serve primarily as validation in humans for the first time. For the relation between ncRNA expressions and ncRNA promoter methylation, this study serves an exploratory role, identifying ncRNA promoters of interest for further investigation using experimental systems. This study is further limited by the heterogeneity of samples and the relatively low sample sizes. Due to working with autopsy samples, factors such as age, health status prior to death, causes of death, time between death and autopsy, and time between autopsy and sample preservation, are uncontrollable and introduce variation in the data, limiting the statistical power of our analyses. We do not expect these limitations to introduce bias into our analyses or conclusions, however, because these factors impact both diabetic and non-diabetic cohorts.

**Fig. 4:**
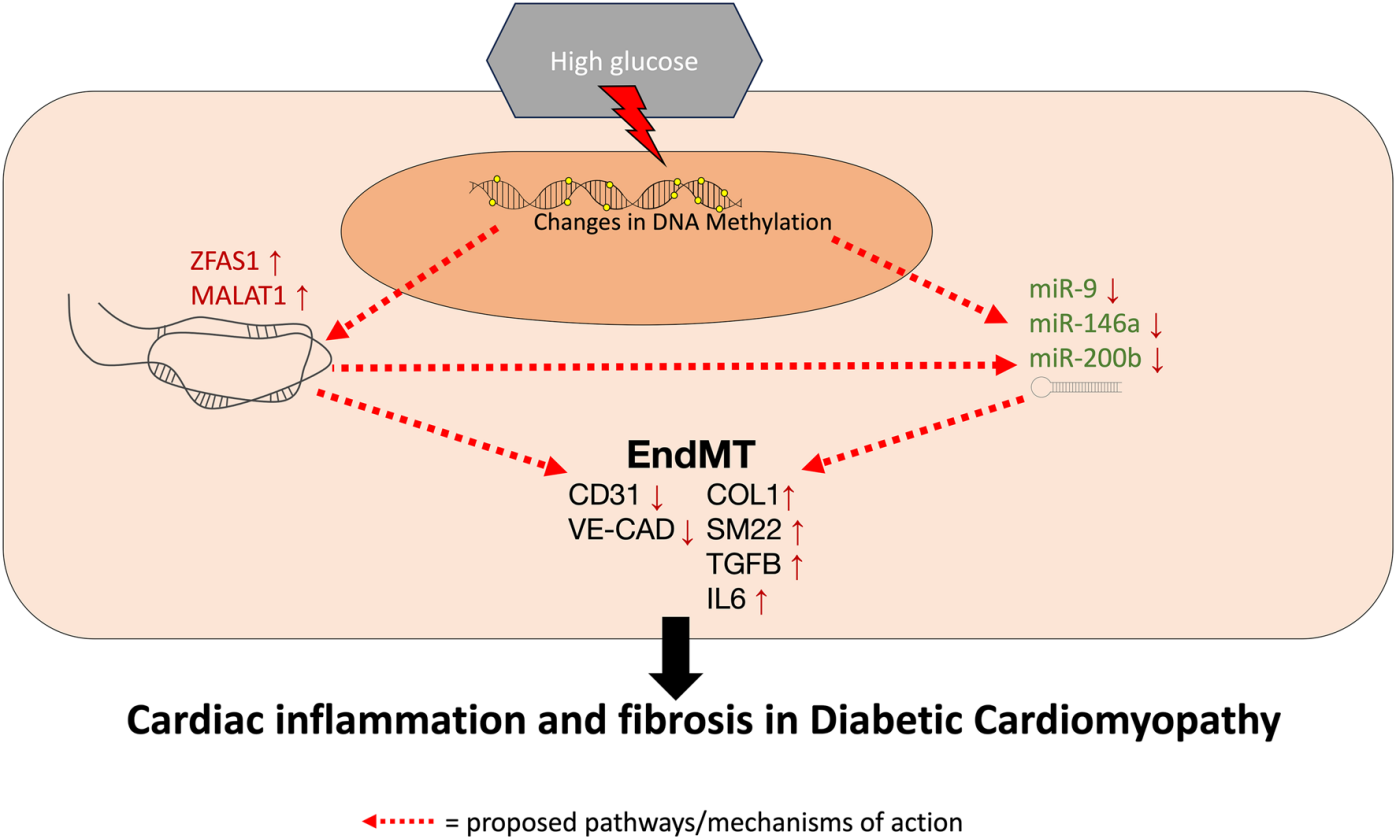
A schematic outlining the proposed mechanisms through which hyperglycemia promotes EndMT in DCM. The proposed mechanism involves high glucose inducing changes in the promoter methylations of key ncRNAs, leading to altered expressions, and resulting in EndMT in DCM. miRNAs are further modulated by lncRNAs, which may act as miRNA sponges, or may target different forms of epigenetic modifications to the genetic loci of miRNAs, such as the suppression of miR-9 by ZFAS1-guided PRC2-mediated histone methylation

In summary, we have used autopsy tissues to verify the occurrence of EndMT in DCM in human hearts. We have further shown that changes in EndMT-related ncRNAs follow the same patterns in human hearts as they do in cell culture and in animal models. Finally, we demonstrated the potential the relation between promoter methylation and the expression levels of EndMT-related ncRNAs. We found that while DNA methylation likely plays a role in the regulation of ZFAS1 and miR-200b, it does not appear to be the main regulator of miR-9 in the heart in DCM. These observational findings validate our previous works and warrant further investigation using experimental models. Overall, this study reinforces the role of epigenetically regulated EndMT in DCM and underscores the complexities and importance of the interactions between different types of epigenetic regulators. We propose a high glucose-induced regulatory axis for EndMT in DCM, shown in Fig. 4. We recognize that the proposed regulatory axis is not fully demonstrated in the current study, but it is based on our solid body of work in this area.

We further recognize that the current study may be limited by sample sizes and variation in autopsy samples. Further experimental studies will allow us to fully elucidate the regulation of ncRNAs by DNA methylation in cardiac ECs in DCM.

### Supplementary Information

**Additional file 1: Table S1.** Complete patient data.

**Additional file 2: Table S2.** Spearman’s Correlation analysis for age/eGFR and genes of interest.

## Data Availability

The datasets used and/or analysed during the current study are available from the corresponding author on reasonable request.

## Abbreviations

DCM: Diabetic cardiomyopathy
DNMT: DNA methyltransferase
EC: Endothelial cell
EndMT: Endothelial-to-mesenchymal transition
HDAC: Histone deacetylase
lncRNA: Long non-coding RNA
miRNA: MicroRNA
ncRNA: Non-coding RNA
RT-qPCR: Reverse transcription quantitative polymerase chain reaction
