# Supplementary material for: Non-coding RNA-mediated endothelial-to-mesenchymal transition in human diabetic cardiomyopathy, potential regulation by DNA methylation
Source: Cardiovasc Diabetol. 2023 Nov 3;22:303. doi: 10.1186/s12933-023-02039-4 (PMC10625293; doi:10.1186/s12933-023-02039-4)
Supplement: Supplementary file 2 — Additional file 2: Table S2. Spearman’s Correlation analysis for age/eGFR and genes of interest. [file 12933_2023_2039_MOESM2_ESM.docx]

| **Table S2.** Spearman's Correlation analysis for age/eGFR and genes of interest | | | | | | | | | |
| --- | --- | --- | --- | --- | --- | --- | --- | --- | --- |
|  | |  | |  | | **Spearman's rho** | | **p** | |
| Age |  | - |  | PECAM1 |  | -0.177 |  | 0.294 |  |
| Age |  | - |  | CDH5 |  | -0.123 |  | 0.516 |  |
| Age |  | - |  | COL1A1 |  | 0.151 |  | 0.435 |  |
| Age |  | - |  | TAGLN |  | 0.013 |  | 0.950 |  |
| Age |  | - |  | TGFB1 |  | 0.197 |  | 0.368 |  |
| Age |  | - |  | IL6 |  | 0.110 |  | 0.525 |  |
| eGFR |  | - |  | PECAM1 |  | -0.230 |  | 0.171 |  |
| eGFR |  | - |  | CDH5 |  | -0.144 |  | 0.445 |  |
| eGFR |  | - |  | COL1A1 |  | -0.032 |  | 0.871 |  |
| eGFR |  | - |  | TAGLN |  | -0.311 |  | 0.115 |  |
| eGFR |  | - |  | TGFB1 |  | 0.361 |  | 0.092 |  |
| eGFR |  | - |  | IL6 |  | 0.090 |  | 0.601 |  |
|  | | | | | | | | | |
|  | | | | | | | | | |
